# Supplementary material for: Statistical modeling based on structured surveys of Australian native possum excreta harboring Mycobacterium ulcerans predicts Buruli ulcer occurrence in humans
Source: eLife. 2023 Apr 14;12:e84983. doi: 10.7554/eLife.84983 (PMC10154024; doi:10.7554/eLife.84983)
Supplement: Supplementary file 1. [file elife-84983-supp1.docx]

**Supplementary File 1:** Impact on model performance by expanding the exposure window

| **Model** | **Exposure window**  **(days)** | **AUC** | **Proportion of BU cases in top 20% predicted case-containing mesh blocks** |
| --- | --- | --- | --- |
| MP-MP-scat | 101-171 | 0.66 | 0.34 |
| MP-MP-scat | 61-277 | 0.61 | 0.25 |
| MP-G-scat | 101-171 | 0.75 | 0.67 |
| MP-G-scat | 61-277 | 0.70 | 0.50 |
| MP-MP-2018-cases | 101-171 | 0.55 | 0.28 |
| MP-MP-2018-cases | 61-277 | 0.52 | 0.25 |
| MP-G-2019-cases | 101-171 | 0.50 | 0.17 |
| MP-G-2019-cases | 61-277 | 0.50 | 0.19 |
